# Research on the comparison of the demethylvancomycin’s diffusion–deposition characteristics in the ocular solid tissues of sustained subtenon drug delivery with subconjunctival injection

**DOI:** 10.1080/10717544.2016.1230904

**Published:** 2017-02-03

**Authors:** Yi-Qin Duan, Ye-Zhen Yang, Xue-Tao Huang, Ding Lin

**Affiliations:** 1 Aier School of Ophthalmology, Central South University, ChangSha, P. R. China and; 2 Changsha Aier Eye Hospital, ChangSha, P. R. China

**Keywords:** Sustained subtenon drug delivery, demethylvancomycin, intraocular solid tissues, diffusion–deposition, area under the curve

## Abstract

*Purpose*: To compare the demethylvancomycin’s diffusion–deposition characteristics in the ocular solid tissues of sustained subtenon drug delivery with subconjunctival injection.

*Method*: Sixty adult white rabbits were randomly assigned to the subtenon drug delivery group and the subconjunctival injection group. The subtenon drug delivery group was continuously infused demethylvancomycin to the subtenon of rabbits. The subconjunctival injection group was injected demethylvancomycin to the subconjunctival of rabbits. Cornea, iris and sclera were collected for high-performance liquid chromatography analyses to determine drug concentrations at one hour, three hours, six hours, 12 h and 24 h of drug administration. WinNonlin 6.3 was used to calculate the parameters of cumulative area under the curve (AUC_cum_) of demethylvancomycin.

*Results*: The peak levels of demethylvancomycin concentration of the subtenon drug delivery group and the subconjunctival injection group were 92.406 ± 21.555 and 51.778 ± 14.001 μg/g in cornea, 28.451 ± 10.229 μg/g and 42.271 ± 27.291 μg/g in iris, 153.166 ± 51.738 μg/g and 57.423 ± 18.480 μg/g in sclera. The differences of concentrations between the two groups in cornea and sclera were statistically significant (*F* = 487.775, *p* < 0.001; *F* = 132.748, *p* < 0.001). The difference in iris was not statistically significant (*F* = 4.848, *p* = 0.064). The maximum of AUC_cum_ of the subtenon drug delivery group and the subconjunctival injection group was 1808.23 h * μg/g and 273.73 h * μg/g in cornea, 489.12 h * μg/g and 216.16 h * μg/g in iris and 2166.34 h * μg/g and 392.57 h * μg/g in sclera at 24 h of drug administration.

*Conclusion*: The sustained subtenon drug delivery had a better drug permeability and accumulation in the intraocular solid tissue compared to subconjunctival injection, which demonstrated it was probably a promising and effective approach for treating posterior segment diseases and endophthalmitis.

## Introduction

There are four major routes for ocular drug delivery including topical application, periocular drug delivery, intraocular injection and systemic administration. Topical application is unable to reach a therapeutic concentration in intraocular tissues for corneal barrier (Ahmed, 2003; Barar et al., 2008). Intravenous administration was associated to systemic side effects and low therapeutic concentration for the blood–eye barrier in the eyes (Hughes et al., 2005; Janoria et al., 2007). Intraocular injection can bypass blood–eye barrier and attain a high intraocular drug concentration, however, repeated injection might cause infectious endophthalmitis, retinal toxicity and increase other unpredicted risks (Wu et al., 2008; Penha et al., 2011; Cristina et al., 2012; Agard et al., 2015). Periocular drug delivery or transscleral drug delivery has been explored as a potentially promising and effective route for posterior segment drug delivering. Studies have shown the sclera to be permeable to betamethasone, oligonucleotides, albumin and the anti-angiogenic molecules (Okabe et al., 2003; Shuler et al., 2004; Cruysberg et al., 2005; Anderson et al., 2008). However, the release systems including gel formulations, polymeric implants, microspheres, liposomes and ocular inserts are limited to bio-degradation and cannot be precisely controlled (Bourges et al., 2006; Gupta et al., 2007; Mathurm & Gilhotra, 2011; Li et al., 2012; Kalam et al., 2016; Yousry et al., 2016).

Demethylvancomycin or demethyl, norvancomycin weighing 1435.19 Da is a derivative of vancomycin, that is a methyl CH3– removed from vancomycin, which shares a similar heptapeptide trunk and varies in the number, type, and placement of sugar substituents attached to the peptide nucleus (Hunt & Vernon, 1981; Nagarajan, 1993). Demethylvancomycin has the similar effectiveness to vancomycin that has been considered as first-line antibiotics to mostly Gram-positive bacteria (Yan et al., 2000).

In our study, an auto-infusion pump was used to deliver demethylvancomycin in a small amount to the subtenon of rabbits by automatic, timing and quantitative control. Cornea, iris and sclera were collected for high-performance liquid chromatography analyses to determine the concentration of demethylvancomycin and WinNonlin 6.3 was used to calculate the parameters of cumulative area under the curve (AUC_cum_) to access demethylvancomycin diffusion–deposition in intraocular solid tissues.

## Materials and methods

### Animal

Sixty adult New Zealand white rabbits weighing 2.0–2.5 kg with animal health immunization certificates were provided by an authorized animal center of The Third Xiangya Hospital of Central South University and were kept in the individual cages under standardized conditions. Rabbits were treated in accordance with guideline and statements of the Association for Research in Vision and Ophthalmology. This study was approved by Animal Ethical Committee of Central South University and Changsha AIER Eye Hospital. The rabbits were intramuscularly anesthetized with the compound preparation of xylazine for 0.1 ml/kg (Lu-Mian-Ning II, Zhong-Lian Corp, Hubei, China). 0.5% proparacaine hydrochloride (Alcaine, Alcon-Couvreur, Puurs, Belgium) was used for topical anesthesia.

### Study groups

All rabbits were randomly assigned to the subtenon drug delivery group and the subconjunctival injection group. Each group of rabbits was then randomly assigned to subgroups of five sequent time points: one hour, three hours, six hours, 12 h and 24 h of drug administration. Six rabbits at each time point were sacrificed and the right eyes were enucleated. Cornea, iris and sclera were collected for high performance liquid chromatography analyses (LG-20AT) to determine the concentration of demethylvancomycin. The extreme values were deleted to avoid the bias errors in data processing. The same number of new samples was added to be remeasured. The records that had a good balance of mean and standard deviation were kept.

### Surgery procedure

**Figure 1.**
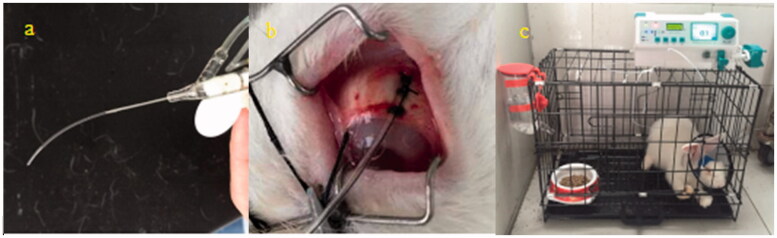
A polyvinyl chloride tube was fixed with the suture on the sclera of rabbit (a,b); another end of the tube was connected with an auto-infusion pump to infuse the drug to the subternon (c).

1. The subtenon drug delivery group: rabbits under well-anesthetized conditions, the conjunctival sac of right eyes was sterile and eyedrops are instilled for topical anesthesia. Eyelid speculum was inserted to expose the bulbar conjunctiva. The supertemporal bulbar conjunctiva apart 2 mm from the edge of cornea was cut open. After blunt separation of the fascia between Ternon capsule and sclera, a polyvinyl chloride tube in length of 3–4 cm was fixed on the surface of the sclera with the suture. The conjunctival incision was sewed up and the tube was taped on the head of rabbit. Another end of the tube was connected with an auto-infusion pump (BYZ-810T, BI-YANG Corp, China). 0.3 ml demethylvancomycin of 20 mg/ml was in bolus infused to the subtenon as the initial drug load and continuously infused with the flow rate of 0.1 ml/h to the subtenon of rabbits. All the rabbits were kept in individual cages and provided with food and water during drug administration (see Figure 1).
2. The subconjunctival injection group: rabbits under well-anesthetized conditions the conjunctival sac of right eyes was sterile and eyedrops are instilled for topical anesthesia. 0.3 ml demethylvancomycin 20 mg/ml was injected to the subconjunctival.

### Sample collection

Six rabbits at each time point were sacrificed and the right eyes were enucleated immediately. 0.9% saline was used to wash the eyeballs to avoid residual drug adhering to the surface of eyeballs. Cornea, iris and sclera adjacent to drug administration area were collected and weighed. Samples were stored at −20 °C refrigerator before analysis.

### Analytical procedures

1. *Sample preparation*: Thawed at room temperature, the samples were tissue homogenated, 200 μl of the supernatant was extracted and mixed with 68 μg/ml demethylvancomycin 20 μg and 24% perchloric acid 20 μg. The mixed liquor was vibrated for three minutes later placed in 4 °C refrigerator for 10 min then centrifuged at 15 000 r/min for 10 min. The supernatant layer was collected and added with 10 μg · ml^−1^ NaOH then vibrated for one minute and centrifuged at 15 000 r/min for 10 min. Twenty microliters of supernatant was extracted to be used for analyzing drug concentration of specimens.
2. *Demethylvancomycin concentration analysis*: High performance liquid chromatography (LC-20AT, Shimadzu, Kyoto, Japan) was used to analyze demethylvancomycin concentration in vitreous humor of rabbits. The detailed parameters were as follows: analytical column: InertSustain® C18 (5 μm × 4.6 mm × 250 mm, GL Sciences Inc., Tokyo, Japan), mobile phase: potassium dihydrogen phosphate–methanol of 30 mmol/l (PH: 2.70, acetonitrile = 88:12), flow velocity 0.8 ml/min, detection wavelength 330 nm, temperature 40 °C.

### Statistical analysis

Statistical Package for Social Science (SPSS; SPSS Inc., Chicago, IL) 18.0 was used for performing statistical analysis of the data. One-way analysis of variance was used to compare the overall difference between the subtenon drug delivery group and the subconjunctival injection group. Independent sample *t*-test was used to compare the differences between the two groups at each time point. The significance level *α* was 0.05.

### Cumulative area under the curve calculation

Pharmacokinetics software WinNonlin 6.3 was used to calculate the parameters of AUC_cum_. Demethylvancomycin concentration–time data were analyzed by noncompartmental analysis (NCA) based on statistical moment theory. Linear Trapezoidal Method was applied to the calculation of AUC_cum_. The calculation formulas are as follows:

(1) $${\mathrm{AUC}}_{0-\infty }=\int_{0}^{\infty }c\left(t\right)\mathrm{dt}$$

(2) $${\mathrm{AUC}}_{0-\infty }={\mathrm{AUC}}_{0-t}+{\mathrm{AUC}}_{t-\infty }$$

(3) $${\mathrm{AUC}}_{0-\infty }={\mathrm{AUC}}_{0-t}$$

(4) $${\mathrm{AUC}}_{0-t}=\int_{0}^{t}c\left(t\right)\mathrm{dt}=\sum \left(C_{t}+C_{t-1}\right)\left(t_{t}-t_{t-1}\right)/2$$

(5) $${\mathrm{AUC}}_{\mathrm{CUM}}={\mathrm{AUC}}_{0-t}=\sum \left(C_{i}+C_{i-1}\right)\left(t_{i}-t_{i-1}\right)/2\left(i=0:t\right)$$

AUC_0–∞_ is the AUC from zero to the infinite time point. AUC_0–_*_t_* is the AUC from zero to the last time point. AUC*_t_*_–∞_ is the AUC from the last time point to the infinite time point. AUC_cum_ is the cumulative AUC at sequent time points. *C* is the demethylvancomycin concentration in intraocular solid tissues at sequent time points. *t* is the last time point of drug administration. *i* is the random time point from zero to the last time point.

The calculus method is used for the calculation of AUC_0–∞_ on zero-order moment (Formula 1). In our study, there was a steady-state drug concentration during the subtenon drug delivery. No elimination phase was on demethylvancomycin concentration–time curve of the subtenon drug delivery group, thus AUC*_t_*_–∞_ equals to zero and Formula 2 can be converted to Formula 3. AUC_cum_ is represented by AUC_0–_*_t_* (formula 5) and AUC_0–_*_t_* is calculated with Linear Trapezoidal Method (Formula 4).

## Results

**Chart 1.**
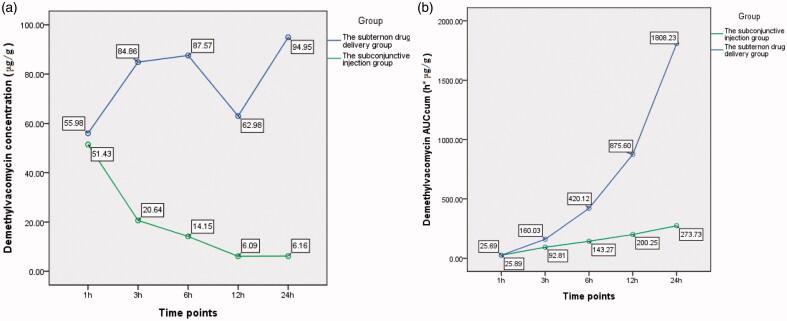
(a) The curve of concentration–time of demethylvancomycin in cornea tissue sequent time points of two groups. (b) The curve of AUC_cum_–time of demethylvancomycin in cornea tissues at sequent time points of two groups.

The difference of demethylvancomycin concentrations in cornea between the two groups was statistically significant (*F* = 487.775, *p* < 0.001; analysis of variance). The differences at sequent time points were statistically significant at three hours, six hours, 12 h and 24 h after drug administration (*t*3 = 18.73, *p* < 0.001; *t*6 = 15.884, *p* < 0.001; *t*12 = 5.504, *p* = 0.002; *t*24 = 9.862, *p* < 0.001; *t*-test) (see Table 1). The peak level of demethylvancomycin concentration in cornea tissue of the subtenon drug delivery group was 94.95 μg/g after continuously infused for 24 h and the peak level of the subconjunctival injection group was 51.43 μg/g at one hour after subconjunctival injection (see Chart 1a). The AUC_cum_–time curve of demethylvancomycin in cornea tissue of the subtenon drug delivery group was in a steep ascending trend, while the AUC_cum_–time curve of the subconjunctival injection group was in a flat ascending trend. The maximum values of AUC_cum_ of the subtenon drug delivery group and the subconjunctival injection group were 1808.23 h*μg/g and 273.73 h*μg/g respectively after 24 h drug administration (see Chart 1b).

**Table 1.** The variation of demethylvancomycin concentration in corneal tissue at sequent time points of the two groups (units: μg/g).

	1 h	3 h	6 h	12 h	24 h	
Subternon capsular infusion group	51.386 ± 18.653	82.951 ± 7.471	90.442 ± 9.170	63.031 ± 25.149	92.406 ± 21.555	*F* = 487.775
Subconjunctive injection group	51.778 ± 14.001	0.45 ± 0.26	20.074 ± 4.066	6.116 ± 3.983	6.132 ± 2.473	*p* < 0.001
	*t*1 = 0.013	*t*3 = 18.73	*t*6 = 15.884	*t*12 = 5.504	*t*24 = 9.862	
	*p* = 0.990	*p* < 0.001	*p* < 0.001	*p* = 0.002	*p* < 0.001	

Note: *F* value was the testing value of one-way analysis of variance to compare the overall difference between the two groups, *t* values were the testing values of two independent sample *t*-tests to compare the difference at each sequent time point.

**Chart 2.**
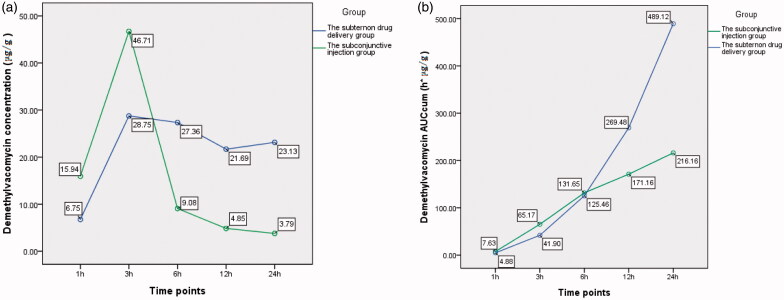
(a) The curve of concentration–time of demethylvancomycin in iris tissues at sequent time points of two groups. (b) The curve of AUC_cum_–time of demethylvancomycin in iris tissues at sequent time points of two groups.

The difference of demethylvancomycin concentrations in iris between the two groups was not statistically significant (*F* = 4.848, *p* = 0.064; analysis of variance). But the differences at sequent time points were statistically significant at six hours, 12 h and 24 h after drug administration (*t*6 = 4.191, *p* = 0.002; *t*12 = 5.818, *p* < 0.001; *t*24 = 2.616, *p* = 0.047; *t*-test) (see Table 2). The peak value of demethylvancomycin concentration in iris tissue of the subtenon drug delivery group was 28.75 μg/g and the subconjunctival injection group was 46.71 μg/g at three hours after drug administration. The demethylvancomycin concentrations in iris tissue of the subtenon drug delivery group was higher than the subconjunctival injection group from one hour to three hours, but became lower from six hours to 24 hours after drug administration (see Chart 2a). The AUC_cum_–time curve of demethylvancomycin in iris tissue of the subtenon drug delivery group was in a steep ascending trend and the subconjunctival injection group was in a flat ascending trend. The values of AUC_cum_ of the subtenon drug delivery group were lower than the subconjunctival injection group from one hour to six hours, while became higher from six hours to 24 h of drug administration adversely. The maximum values of AUC_cum_ of the subtenon drug delivery group and the subconjunctival injection group were 489.12 h*μg/g and 216.16 h*μg/g respectively at 24 h of drug administration (see Chart 2b).

**Table 2.** The variation of demethylvancomycin concentration in iris tissue at sequent time points of the two groups (units: μg/g).

	1 h	3 h	6 h	12 h	24 h	
Subternon capsular infusion group	9.759 ± 8.386	27.258 ± 7.153	28.451 ± 10.229	20.046 ± 6.223	16.664 ± 18.078	*F* = 4.848
Subconjunctive injection group	15.268 ± 4.670	42.271 ± 27.291	9.737 ± 4.238	4.200 ± 2.817	3.333 ± 0.701	*p* = 0.064
	*t*1= −1.488	*t*3= −1.353	*t*6 = 4.191	*t*12 = 5.818	*t*24 = 2.616	
	*p* = 0.171	*p* = 0.209	*p* = 0.002	*p* < 0.001	*p* = 0.047	

Note: *F* value was the testing value of one-way analysis of variance to compare the overall difference between the two groups, *t* values were the testing values of two independent sample *t*-tests to compare the difference at each sequent time point.

**Chart 3.**
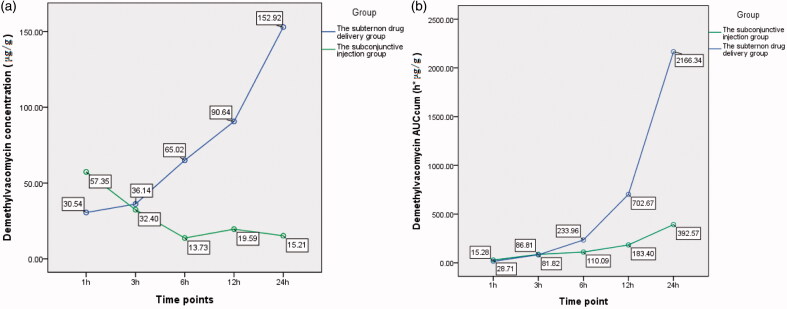
(a) The curve of concentration–time of demethylvancomycin in sclera tissues at sequent time points of two groups. (b) The curve of AUC_cum_–time of demethylvancomycin in sclera tissues at sequent time points of two groups.

The difference of demethylvancomycin concentrations in sclera between the two groups was statistically significant (*F* = 132.748, *p* < 0.001; analysis of variance). The differences at sequent time points were statistically significant at one hour, six hours, 12 h and 24 h after drug administration (*t*1 = 3.489, *p* = 0.006; *t*6 = 5.130, *p* < 0.001; *t*12 = 9.832, *p* < 0.001; *t*24 = 7.217, *p* < 0.001; *t*-test) (see Table 3). The peak value of demethylvancomycin concentration in sclera tissue of the subtenon drug delivery group was 152.92 μg/g after continuously infused for 24 h and the peak value of the subconjunctival injection group was 57.35 μg/g at one hour after subconjunctival injection. The demethylvancomycin concentrations in sclera tissue of the subtenon drug delivery group were higher than the subconjunctival injection group from one hour to three hours after drug administration but were lower from three hours to 24 h (see Chart 3a). The AUC_cum_–time curve of demethylvancomycin in sclera tissue of the subtenon drug delivery group is in a steep ascending trend and the subconjunctival injection group was in a flat ascending trend. The maximum values of AUC_cum_ of the subtenon drug delivery group and the subconjunctival injection group were 2166.34 h*μg/g and 392.57 h*μg/g respectively at 24 h after drug administration. From one hour to three hours the trends of AUC_cum_ curves of the two groups were almost approximate, but from three hours to 24 h the AUC_cum_ curve of the subtenon drug delivery group rose up sharply compared to the subconjunctival injection group (see Chart 3b).

**Table 3.** The variation of demethylvancomycin concentration in scleral tissue at sequent time points of the two groups (units: μg/g).

	1 h	3 h	6 h	12 h	24 h	
Subternon capsular infusion group	30.565 ± 10.070	35.971 ± 10.173	65.457 ± 26.872	90.779 ± 18.942	153.166 ± 51.738	*F* = 132.748
Subconjunctive injection group	57.423 ± 18.480	32.574 ± 12.037	13.702 ± 4.640	19.798 ± 5.703	15.259 ± 6.386	*p* < 0.001
	*t*1= −3.489	*t*3 = 0.606	*t*6 = 5.130	*t*12 = 9.832	*t*24 = 7.217	
	*p* = 0.006	*p* = 0.558	*p* < 0.001	*p* < 0.001	*p* < 0.001	

Note: *F* value was the testing value of one-way analysis of variance to compare the overall difference between the two groups, *t* values were the testing values of two independent sample *t*-tests to compare the difference at each sequent time point.

## Discussion

Nowadays, the major routes for ocular drug delivery include topical application, periocular drug delivery, intraocular injection and systemic administration. Topical application is limited for the factors of cornea barrier, lacrimal drainage, absorption of conjunctiva and the turnover of tear film. It is estimated that less than 5% of the applied drug can reach the targeted tissues due to the extensive precorneal factors (Ahmed, 2003; Barar et al., 2008). Intravenous administration associated to systemic side effects and low therapeutic concentration for the blood–eye barrier in the eyes (Hughes et al., 2005; Janoria et al., 2007). Intraocular injection offers the most direct route to attain the high drug concentration bypassing the blood ocular barrier and the blood aqueous barrier, however, the excessive drug concentration may cause retina toxicity and repeated injection is needed due to the rapid drug elimination. Additionally intraocular injections may increase the risks of hemorrhage, endophthalmitis, retinal detachment and cataract (Wu L et al., 2008; Penha et al., 2011; Agard et al., 2015).

Periocular drug delivery or transscleral drug delivery containing subconjunctival, subtenon, retrobulbar and peribulbar administration, is relatively less invasive than intraocular injection and considered as a potentially route for the posterior segment drug delivering. The factors affecting to transscleral drug delivery to posterior segment ocular tissue include diffusion across eyeball wall, active transport in RPE, distribution and clearance by circulation (Ranta & Urtti, 2006; Urtti, 2006).

In our experiment, the drug was directly infused to the subtenon space of rabbits via a polyvinyl chloride tube connected with an auto-infusion pump. Drug administration could be controlled with given time and qualities. Consequently, the time duration of drug and sclera contacting with each other was long enough to maintain a therapeutic and steady drug concentration.

The sclera that is of average 17 cm^2^ surface provides a relatively larger avenue for drug delivery to the inside of the eye compared to the 1 cm^2^ surface of the cornea (Olsen et al., 1998). The sclera by virtue of its large surface area and relatively high permeability may provide an ideal approach for delivering drugs to posterior segment tissues (Borcherding et al., 1975). The sclera permeability is dependent on the molecular weight of solutes that the drugs of larger molecular radius require longer time to cross the sclera (Ambati et al., 2000). Kau and colleagues demonstrated the sclera permeabilities (Ktrans) were 6.66 ± 1.46 × 10(−7) for vancomycin, 3.90 ± 0.59 × 10(−7) for polymyxin B and 1.89 ± 0.21 × 10(−6) for penicillin G. The drug molecules crossing the sclera in passive diffusion first accumulate in the collagen tissues then diffuse into the aqueous humor and the vitreous in the process of re-release (Kau et al., 2005). For that reason, the collagen tissue drug concentrations of eye could be more useful for predicting the drug efficacy instead of the fluid tissue.

The main corneal barriers are the lipophilic epithelium layers with its tight junctions, and the hydrophilic stroma that are the rate-limiting barriers for lipophilic drugs absorption (Robinson, 1993; Rabinovich-Guilatt et al., 2004). The principal components of the scleral stroma are collagen fibers, accounting for 75% of the scleral dry weight with type I being the major collagen type. Collagen fibers are the branched and interwoven array in the sclera but the lamellar and orderly array in the central cornea (Borcherding et al., 1975). In our study, demethylvancomycin concentrations of the subtenon drug delivery group in cornea and sclera are higher than the subconjunctival injection group. The difference was statistically significant (*F* = 487.775, *p* < 0.001; *F* = 132.748, *p* < 0.001). It demonstrated that by the virtue of the relatively uniform collagen structure of sclera, more demethylvancomycin molecules were accumulated in cornea and sclera through the continuous subtenon drug infusion. Although the difference between the two groups in iris was not statistically significant (*F* = 4.848, *p* = 0.064). Even demethylvancomycin concentration in iris of the subconjunctival injection group was higher than the subtenon drug delivery group at three hours of drug administration. With the time increasing, demethylvancomycin concentrations in iris of the subtenon drug delivery group became higher than the subconjunctival injection group. The differences between two groups at sequent time points were statistically significant at six hours, 12 h and 24 h of drug administration (*t*6 = 4.191, *p* = 0.002; *t*12 = 5.818, *p* < 0.001; *t*24 = 2.616, *p* = 0.047). The iris consists of five layers including anterior border layer, stroma, muscular layer, anterior and posterior iris epithelia. Stroma makes up most of the iris that is rich of spongy mixture of blood vessels, collagen, fibroblast, melancytes and nerve fibers. Our results demonstrated that the demethylvancomycin’s diffusion–deposition characteristics of the three intraocular solid tissues were different, which may be due to the reason that iris contains a large amount of blood vessels but little vessels are in cornea and sclera. The demethylvancomycin concentration in iris of the subconjunctival injection group was higher than the subconjunctival injection group at three hours of drug administration. The possible reason may be related to the different drug administration areas of the subtenon drug delivery group and the subconjunctival injection group. The administration area of subconjunctival injection was located on the anterior part of the sclera while subtenon drug delivery was on the posterior part of the sclera, which means the drugs administrated by subconjunctival injection were feasible to diffuse into the iris tissue by shorter routes and the diffusing process might take a shorter time than the subtenon drug delivery, but the advantage phenomenon disappeared as the time increased.

In our study, there was a steady-state drug concentration trend during the subtenon drug delivery. No elimination phase was on the demethylvancomycin concentration–time curve of the subtenon drug delivery group, the demethylvancomycin concentration–time curve did not match the compartmental model of pharmacokinetics. So we applied NCA to analyze the demethylvancomycin concentration–time data based on statistical moment theory. AUC_cum_ represents the accumulative effect of drugs in tissues by calculating the area size under the concentration–time curve. It provides a more intuitive way to present the real situation of demethylvancomycin diffusion–deposition in the ocular solid tissues. In this study the AUC_cum_–time curve of demethylvancomycin of the subtenon drug delivery group in cornea, iris and sclera was in a steep ascending trend but the AUC_cum_–time curve of the subconjunctival injection group was in a flat ascending trend. Additionally, the maximum values of AUC_cum_ of the two groups both appeared at 24 h of drug administration. It well demonstrated the real effect of demethylvancomycin diffusion–deposition in ocular solid tissue of the subtenon drug delivery group was superior to the subconjunctival injection group.

There were some limitations in this study. It may be related to the small sample size at each time point and the individual variation of rabbits. In addition, the demethylvancomycin injection used in our study was the suspension which might block the tube in long-term drug administration in rabbits. Thirdly, the tube could fall off with the scratching of rabbits or be bitten off by rabbits even though they were placed in plastic neck scarf. In the further study, we may substitute demethylvancomycin for another antibiotic with smaller molecular radius and better water solubility. We should modify the defects of study design and expand the sample sizes of rabbits to decrease the experimental and bias errors.

In conclusion, although demethylvancomycin that we used in this study is a sort of macromolecular drugs with poor permeability crossing the sclera, it was feasible to deliver demethylvancomycin in a little amount to the subtenon space of rabbits by automatic, timing and quantitative control via an auto-infusion pump. Demethylvancomycin had a better permeability and more accumulation in the intraocular solid tissue and consequently could reach a more effective therapeutic concentration in the intraocular tissues. It indicated subtenon drug delivery is a promising and effective approach for the ocular delivery.
